# Distribution of ultrasonic radiofrequency signal amplitude detects lipids in atherosclerotic plaque of coronary arteries: an ex-vivo study

**DOI:** 10.1186/1476-7120-6-18

**Published:** 2008-05-09

**Authors:** Hisao Hara, Taro Tsunoda, Naohiko Nemoto, Itaru Yokouchi, Masaya Yamamoto, Tsuyoshi Ono, Masao Moroi, Makoto Suzuki, Kaoru Sugi, Masato Nakamura

**Affiliations:** 1 Division of Cardiovascular Medicine, Toho University Ohashi Medical Center, Tokyo, Japan

## Abstract

**Background:**

Accumulation of lipids within coronary plaques is an important process in disease progression. However, gray-scale intravascular ultrasound images cannot detect plaque lipids effectively. Radiofrequency signal analysis could provide more accurate information on preclinical coronary plaques.

**Methods:**

We analyzed 29 zones of mild atheroma in human coronary arteries acquired at autopsy. Two histologic groups, i.e., plaques with a lipid core (group L) and plaques without a lipid core (group N), were analyzed by automatic calculation of integrated backscatter. One hundred regions of interest were set on the target zone. Radiofrequency signals from a 50 MHz transducer were digitized at 240 MHz with 12-bit resolution. The intensity of integrated backscatter and its distribution within each plaque were compared between the two groups.

**Results:**

Although the mean backscatter was similar between the groups, intraplaque variation of backscatter and backscatter in the axial direction were larger in group L than in group N (p = 0.02). Conventional intravascular ultrasound showed extremely low sensitivity for lipid detection, despite a high specificity. In contrast, a cut-off value>32 for the total variance of integrated backscatter identified lipid-containing plaque with a high sensitivity (85%) and specificity (75%).

**Conclusion:**

Compared with conventional imaging, assessment of the intraplaque distribution of integrated backscatter is more effective for detecting lipid. As coronary atheroma progresses, its composition becomes heterogeneous and multi-layered. This radiofrequency technique can portray complex plaque histology and can detect the early stage of plaque progression.

## Introduction

The accumulation of lipids within coronary plaques is an important process in the progression of atherosclerosis and plaque vulnerability. Early detection of lipid-rich plaque could be useful for predicting future coronary events such as acute myocardial infarction and sudden cardiac death. High-risk plaques have been pathologically categorized as having a large lipid core, a thin fibrous cap, and inflammatory cell infiltration [1,2]. However, *in vivo *tissue characterization using available tools is still challenging in the clinical setting. Intravascular ultrasound (IVUS) is a promising imaging modality for the evaluation of coronary artery disease including atheroma [3]. Analysis of two-dimensional gray-scale IVUS images is widely used for quantitative assessment of lesions, but it is inadequate for investigating plaque histopathology [4]. Processed IVUS data has the limitation of being unable to discriminate between fatty components and loose connective tissue [5,6]. In contrast, analysis of the unprocessed radiofrequency (RF) ultrasound signal can provide more precise information about the architecture of the arterial wall. Many attempts have already been made to characterize tissues by evaluating various parameters of the backscattered signals [7-12]. The present study was designed to determine whether a new system for analysis of the distribution of integrated backscatter (IB) could detect preclinical intraplaque lipid accumulation, corresponding to the initial stage of vulnerable plaque formation.

## Methods

### Specimens and procedures

Ten specimens of human coronary arteries were resected at autopsy from patients without cardiac disease(We used the specimen of eight patients. Femal/male 3/5. Age 76 ± 4 years. Hypertention50%. Diabetis 30%.) and IVUS examination of each specimen was immediately carried out in a water bath. Then the specimens were fixed in 10% buffered formalin for at least 12 hours prior to histopathologic evaluation. Extracellular lipids within plaques were detected by hematoxylin-eosin staining as hypochromic areas that could be distinguished from surrounding acidpholic fibrous tissue. An experienced pathologist arbitrary selected 29 cross-sectional zones of mild atheroma that contained lipids (AHA type IV or V, group L; n = 13), or else only contained fibrous tissue (group N; n = 16).

A co-worker who was experienced in ultrasound studies selected cross-sectional gray-scale IVUS images corresponding with each histological section. Matching of the stained sections and gray-scale images was performed carefully by using the distance from coronary branches as a guide. Another investigator who was blinded to the histopathologic information made a visual diagnosis of the plaque type and analyzed the RF data, including the backscatter intensity and distribution parameters.

### Ultrasound study

A new 3.2 Fr IVUS imaging catheter with a high-frequency 50 MHz transducer (Intra Focus, Terumo Japan) was inserted into the vascular specimens in the water bath. Target segments within the vessels were identified and images were recorded on videotape. Plaques for analysis were selected as regions of mild stenosis with a maximal wall thickness>0.5 mm, no calcification (no acoustic shadowing behind a high-intensity object), and good quality images (no ring or non-uniform rotation distortion artifacts). Visual assessment and RF analysis were performed on a plaque zone that was demarcated by two lines drawn at an angle of 45 degrees from the center of the lumen, the manually traced intimal surface, and the external elastic lamina.

**Figure 1:**
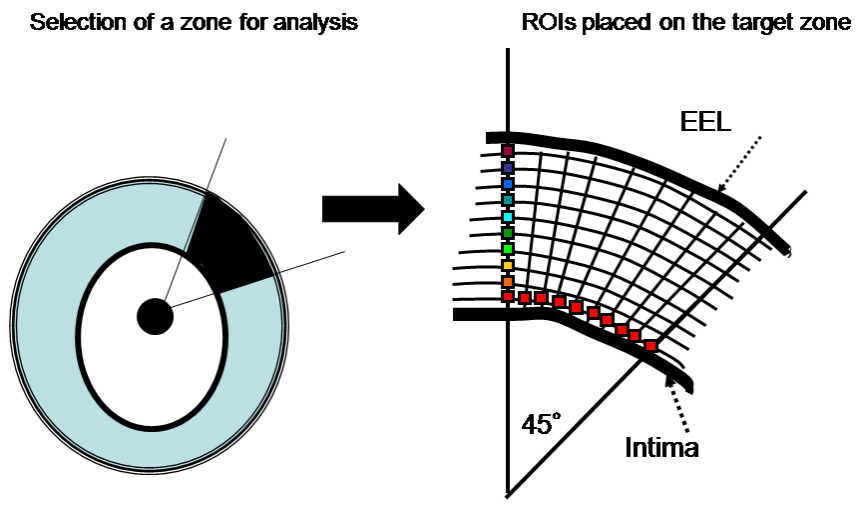
**Target zone selection and ROI placement**. Left: A target zone for analysis was selected at a site where plaque was thicker than 0.5 mm with no calcification and no artifacts. Right: One hundred (10 × 10) ROIs were placed on the target zone, with the innermost 10 and outermost 10 ROIs being carefully placed to exclude the intimal surface and external elastic lamina (EEL), respectively.

**Figure 2:**
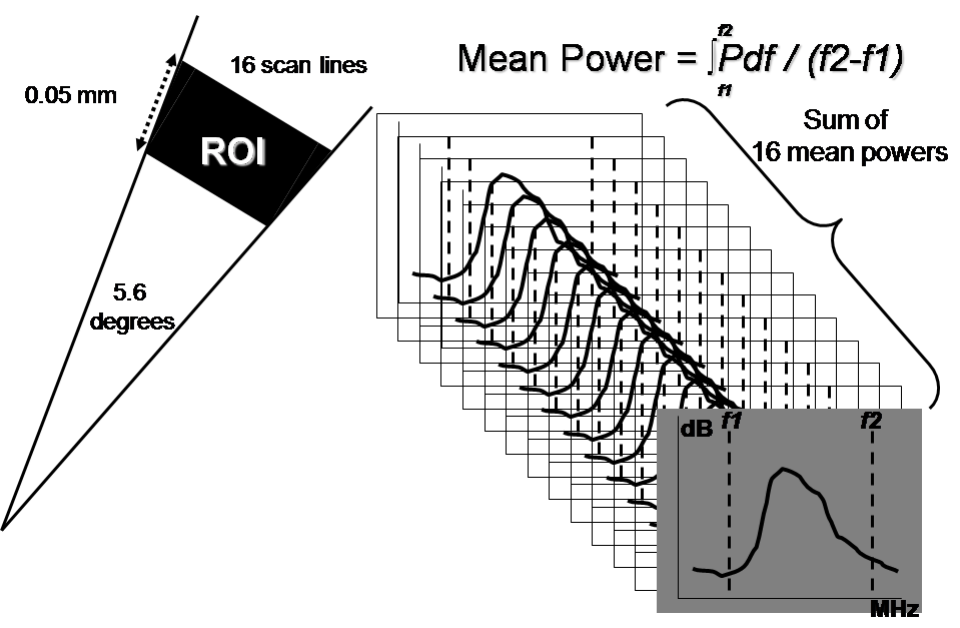
**Radiofrequency signal sampling and calculation of mean power**. Left: Sixteen scan lines from the transducer were placed within each small ROI (an angle of 5.6 degrees), which had a depth of 0.05 mm. Right: The sum of the 16 mean signal powers was calculated as the mean IB value of each ROI.

**Figure 3:**
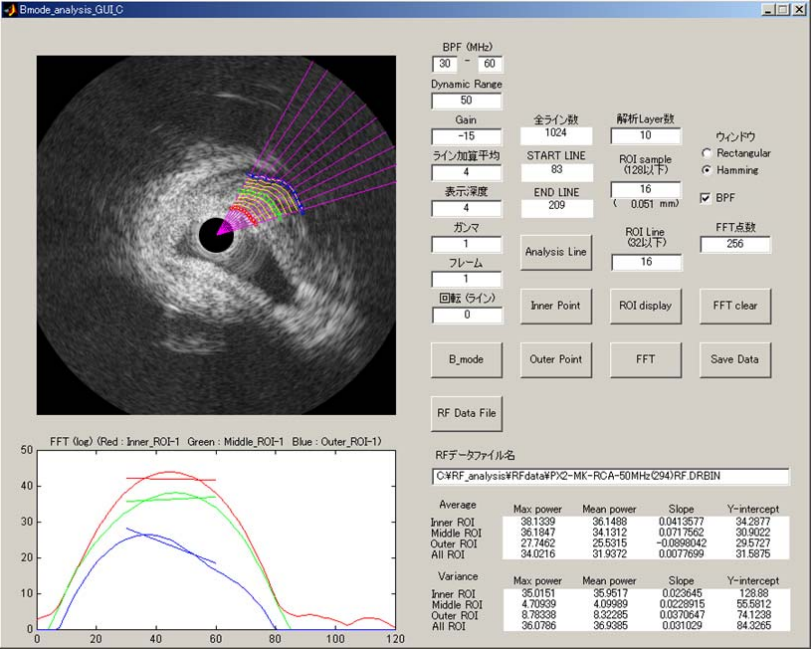
**Computer display of the analysis system**. ROI placement, data acquisition, calculation of IB parameters, and data storage can all be done automatically.

The RF analysis system used in this study was developed by Terumo Japan, and it allows automated placement of regions of interest (ROIs), as well as automated signal acquisition, calculation, and data storage. To analyze the signal intensity distribution, one hundred (10 × 10) very small ROIs were set on the zone of analysis (Fig. 1). The innermost 10 and outermost 10 of these ROIs were carefully placed to exclude the intimal surface and the external elastic lamina, respectively, because of the sudden change in intensity and angle-dependent variation of echo intensity at these boundaries. A total of 16 scan lines from the transducer were placed within each small ROI, which had a depth of 0.05 mm (Fig. 2, left). RF signals within each ROI were digitized at 240 MHz with 12-bit resolution. Then the IB was calculated as the average power (dB) of the signal reflected from the tissues by using fast Fourier transformation. Subsequently, the mean power of the 16 vectors was calculated as the IB power of each ROI (Fig. 2, right). Signals from the 100 ROIs were automatically captured, and the mean IB amplitude and variance among the ROIs in the analysis zone were calculated and displayed by computer. To assess plaque architecture, the variation of IB values in the axial direction and the lateral direction was also calculated using averaged data sets of 10 IB values to assess variance among the 10 consecutive inner to outer lines of ROIs and variance among the 10 consecutive axial lines of ROIs. Offline analysis of the shape factor parameters of the distribution of IB was also performed using RF data stored on the hard disk of the system. Figure 3 shows the analysis system displayed on a laptop computer screen.

### Detectability of plaque lipid cores

The average IB power and the intraplaque distribution of IB were compared between group L and group N. Then IB parameters that could discriminate plaques with a lipid core from those without a lipid core were selected and their cut-off values were calculated. The correspondence between ultrasound findings and histopathology was also assessed. Finally, lipid core detection was compared between this IB technique and conventional GS images by determining the sensitivity and specificity of each method.

### Statistical analysis

Statistical analysis was performed with StatView 5.0 software (SAS Institute, Cary, NC). The two-tailed Student's *t*-test was used to compare parameters between group L and group N. Differences between the shape factor parameters of the data distribution (kurtosis and skewness) were calculated by using Welch's test with SPSS-II version 11.0 software (SPSS Japan). The chi-square test was used to compare the accuracy of plaque lipid detection (sensitivity and specificity) between IB analysis and conventional gray-scale images. Differences were considered significant at p < 0.05 in all analyses.

## Results

### Comparison of RF signals between plaques with and without lipid cores

**Figure 4:**
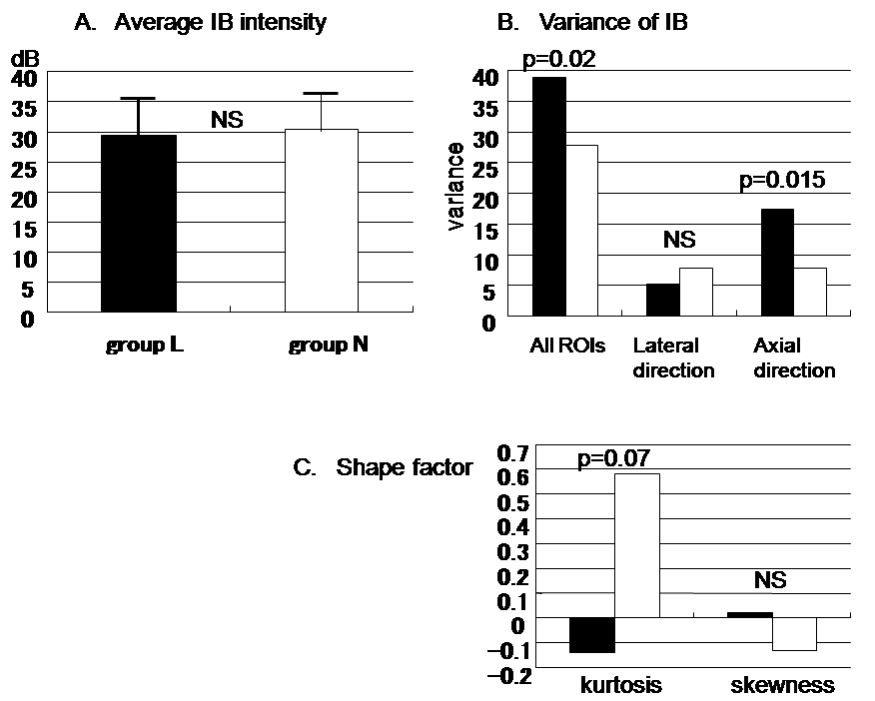
**Average mean IB intensity (A), intraplaque IB distribution (B), and shape factor parameters (C)**. Black bars indicate group L and white bars indicate group N.

Figure 4A shows that the average absolute IB power was similar between groups L and N (29.5 ± 6.5 dB vs. 30.4 ± 6.9 dB). However, the variance of IB among all ROIs was greater in group L than in group N (38.8 vs. 27.8; p = 0.02) (Fig. 4B). The variance of IB in the axial direction was also greater in group L than group N (17.4 vs. 7.8; p = 0.015), while there was no difference of variance in the lateral direction (5.1 in group L and 7.7 in group N).

The shape factor parameters of the IB distribution within each plaque did not show a specific pattern (Fig. 4C). Although there was negative kurtosis in the lipid-rich plaques, unlike the non-lipid-containing plaques (- 0.14 in group L vs. 0.58 in group N; p = 0.07), no difference of skewness was found between the two groups (0.02 in group L vs. – 0.13 in group N).

### Sensitivity of detecting lipid cores

The sensitivity, specificity, positive predictive value, and negative predictive value for detection of plaque lipid cores by the gray-scale and IB methods are shown in Table 1. Cut-off values for the variance of IB among all ROIs and variance in the axial direction were set as 32 and 11, respectively. With gray-scale image estimation, the sensitivity of lipid core detection was extremely low (46%), although the specificity was 75%. Based on analysis of IB, the variance among all ROIs>32 was defined as a parameter for lipid detection (84.6% sensitivity), which was significantly better compared to analysis (p = 0.04) of gray-scale data while maintaining a high specificity. The sensitivity achieved with variance of IB>11 in the axial direction was only 69.2%, and this was not significantly better than gray-scale IVUS.

**Table 1:** Comparison of Intraplaque Lipid Detection Between RF Parameters and Gray-Scale IVUS

	Variance among all ROIs >32	Variance in the axial direction >11	Gray-scale IVUS
Sensitivity (%)	84.6*	69.2	46.2
Specificity (%)	75.0	75.0	87.5
Positive predictive value (%)	73.3	69.2	75.0
Negative predictive value (%)	85.7	75.0	66.7

*P = 0.04 vs. gray-scale IVUS

**Figure 5:**
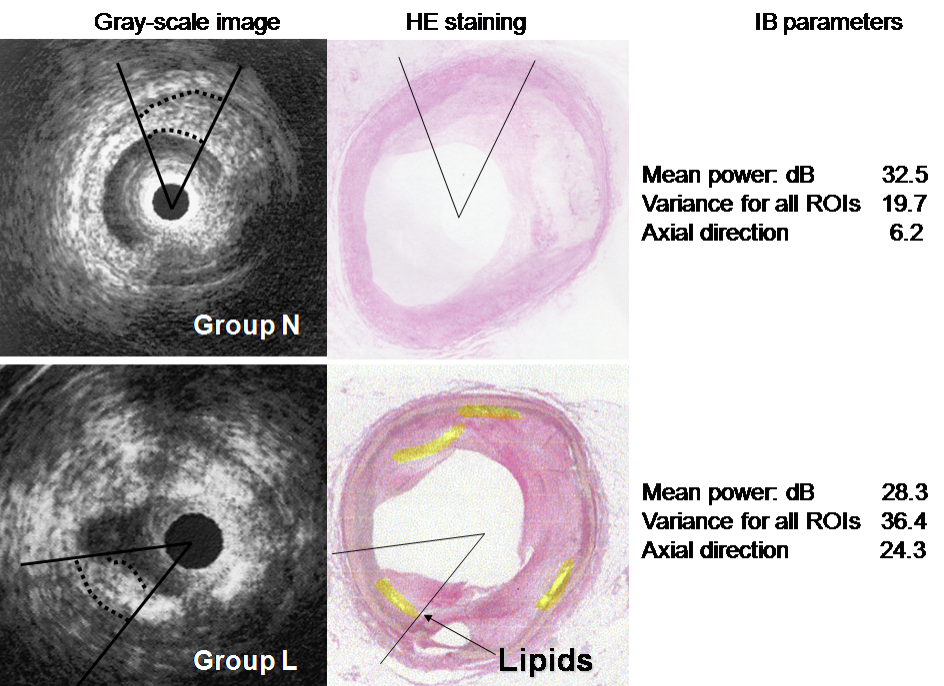
**Analysis of IB parameters discriminates plaque with an early lipid core from plaque without a lipid core using a cut-off value of 32 for the variance of IB.** The gray-scale image fails to display the lipid core (bottom left).

Figure 5 shows an example of successful intraplaque lipid core detection using the IB analysis system.

## Discussion

### Problems with tissue characterization

IVUS is the most useful modality for assessing the coronary artery walls, including atheromatous plaque [13,14]. In addition to quantitative evaluation of plaque size and eccentricity, tissue characterization has become an important issue since the anatomy of vulnerable plaques that are prone to rupture has been clarified [15]. Improvement of conventional IVUS systems has led to better visual estimation of cross-sectional images of plaque, but analysis of gray-scale image data still has limitations with respect to tissue characterization because of large interobserver variability [6], inadequate discrimination between fatty and fibro-fatty tissue [16,17], and lack of standardized parameters due to variation between devices. Many attempts have been made to overcome these problems with limited success [18-20]. Quantitative texture analysis and videodensitometric comparison of plaques with a reference at the adventitia were utilized for the GAIN study [21], which showed that atorvastatin treatment induced a significant increase of the high-echoic component within coronary plaques. This suggested that common lipid-lowering therapy could transform the composition of plaques from fatty to fibrous. Such findings have increased the demand for better tissue characterization that allows discrimination of different plaque components with the use of ultrasound.

### Early detection of intraplaque lipids

Stary et al. updated the histopathologic classification of coronary atherosclerosis [22]. AHA type IV and V lesions are considered to be irreversible atheroma that can progress to unstable plaque (type VI), followed by plaque rupture and thrombotic occlusion of the affected coronary artery. In contrast, AHA type III lesions are defined as preatheroma and mainly consist of loose connective tissue without s lipid core. Histologically, accumulation of extracellular lipids produces a lipid core within atheroma and a large lipid core is a key factor in plaque vulnerability. Therefore, early detection of intraplaque lipids could be important for the prevention of cardiovascular events. In the present study, we defined group L plaques with a lipid core (type IV or V) and group N plaques without a lipid core (type III) according to the results of histological examination.

To compensate for the subjectivity of gray-scale IVUS, analysis of frequency domain signal parameters is widely used. However, previous studies have only compared the IB among different tissues, such as calcificied, dense fibrous, loose fibrous, and lipid-rich plaques, based on the intensity threshold [23,24]. In the present study, we found that the intraplaque distribution of IB was a useful parameter for detection of lipid. In contrast, average IB power alone was not effective for discriminating plaques with a lipid core from plaques without such a core. Likewise, Komiyama et al. previously reported that the sensitivity of lipid core detection was similar between the average IB power and visual analysis of video images [25].

### Variance of IB detects lipid cores

In the present study, we did not standardize the signal intensity of tissues with reference to a perfect reflector, so we did not determine standard IB values. Instead, we investigated the pattern of the intraplaque distribution of IB. As a result, we found greater overall variance of IB and greater variance of IB in the axial direction when plaques contained lipids compared with non-lipidic plaques. Because small ROIs were set on the target zone, the precise distribution of IB within the zone could be determined. The small angle of the scan lines within each ROI (5.6 degrees), and exclusion of signals from the external elastic lamina and the intima could minimize the angle-dependent variability of ultrasound signals [26]. Furthermore, use of 100 ROIs allowed subtle changes of IB to be displayed, representing the heterogenous tissues that compose atheromatous plaques.

A previous histopathologic study demonstrated that atheroma has various components, such as apoptotic debris, microcalcification, and degenerating fibers, in addition to the lipid pool [27]. Heterogeneity of plaque composition, including particle size and structure, may lead to larger IB variance. Additionally, we found a marked increase of IB variance in the axial direction along with the accumulation of intraplaque lipids. These results suggest that the distribution of IB might be able to portray the stratified histologic architecture of plaques, with a deeper lipid core and a superficial fibrous cap.

### Shape factor analysis

Analysis of the shape factor of IB data distribution may provide more precise information for plaque characterization. Picano et al. performed an *in vitro *study of IB signals from the human aorta using a 10 MHz transducer, and evaluated the relation between the shape factor and histologic characteristics [28]. They found lower kurtosis and skewness in atherosclerotic regions compared with normal regions. The present study also revealed a negative mean value of kurtosis in plaques with a lipid core, but not in non-lipidic plaques, suggesting a broader distribution of IB values along with the progression of plaque stage. The similar skewness values for both plaque groups implied that the lipid core coexists with various other higher and lower intensity components of the plaque.

### Sensitivity of lipid core detection

Detection of lipid cores in plaques by conventional gray-scale IVUS shows a very low sensitivity (<50%), as found in both a previous study using a 30 MHz transducer25) and in the present study using a higher frequency (50 MHz) system. A higher sensitivity for lipid core detection compared with conventional gray-scale image analysis is a feature of RF methods [29]. Use of a cut off value for the variance of IB seems to be clinically feasible, and setting a value for variance among all ROIs>32 achieved a sensitivity of 85% while maintaining a high specificity.

### Limitations

Plaque with significant calcification was excluded from this study, although microcalcification is a common finding, even in the early stage of atheroma formation [30]. Ehara et al. reported that spotty calcification was frequently found within the culprit plaques in patients with acute coronary syndrome [31]. Calcium reflects ultrasound and attenuated signals behind calcium deposits interfere with RF-based analysis. For a similar reason, cholesterol crystals within atheroma may also interfere with IB-based assessment [32]. Further investigation of the relation between calcium or cholesterol deposits and coronary events may be necessary in addition to the analysis of plaque composition.

The present study did not define the absolute threshold values for different tissues or develop color mapping. Also, this method could not demonstrate the anatomical localization of the different tissue components. However, simple use of a cut-off value for IB variance may provide useful information for the diagnosis of preclinical coronary artery disease. Although the recently developed IB-IVUS[33,34] and Virtual Histology™[35] systems are user-friendly and are capable of two- and three-dimensional mapping with multi-colored displays, discrimination of neointima and areas behind lipids from hyalinized thrombus and fibrous tissue might still be difficult because their backscatter is similar. As a result, color mapping sometimes portrays inaccurate tissue borders.

### Future Implications

Inhibition of disease progression, as well as plaque stabilization and plaque regression, have recently been demonstrated as beneficial outcomes in large-scale trials of aggressive statin therapy [36,37]. A recent RF study using the IB-IVUS system suggested that stabilization of plaque can be demonstrated as a decrease of the fatty component and an increase of fibrous tissue that enhances plaque integrity [38]. Although IVUS is an invasive examination, our present method could detect changes of plaque composition and architecture after medical interventions such as aggressive cholesterol lowering or modulation of inflammation. It is also possible that *in vivo *analysis of preclinical plaques might allow us to predict future coronary events. Analysis of RF data is a feasible method of coronary plaque characterization that compensates for the poor tissue discrimination, subjectivity, and variation between devices that affect gray-scale IVUS methods. We hope that the discrimination of the organization ingredient is done clinical application of by this method.

## Authors' contributions

All authors read and approved the final manuscript. HH and TT conceived of the study, and participated in its design and coordination. TT participated in the design of the study and performed the statistical analysis. NN, IY, MY and TO made a specimen for light microscopy. MM, MS, KS and MN participated in the design of the study.
